# Sleep problems and childhood adiposity: a cross-sectional study among third-grade students in Shanghai, China

**DOI:** 10.3389/fpubh.2025.1629048

**Published:** 2025-08-14

**Authors:** Shuman Li, Zhe Zhang, Yani Zhai, Liting Chu, Dongling Yang, Chunyan Luo, Lijing Sun

**Affiliations:** Division of Child and Adolescent Health, Shanghai Municipal Center for Disease Control and Prevention, Shanghai, China

**Keywords:** children, sleep, body mass index, waist-to-height ratio, obesity

## Abstract

**Objective:**

To investigate the association between sleep problems and overweight or obesity among third-grade primary school students in Shanghai, China.

**Methods:**

A total of 3,640 students aged 8–10 years were recruited in 2023 using a random cluster sampling method. Sleep problems were assessed using the Children’s Sleep Habits Questionnaire (CSHQ). Anthropometric measurements, including weight, height, and waist circumference, were collected to calculate body mass index (BMI) and waist-to-height ratio (WHtR).

**Results:**

The prevalence of overweight or obesity was 31.7%. The average sleep duration was 9.2 ± 0.6 h, and the mean total CSHQ score was 47.8 ± 7.5. Weekend sleep duration was negatively associated with BMI (β = −0.188, *p* < 0.01). Higher Sleep Disordered Breathing scores were positively associated with BMI (β = 0.246, *p* < 0.01) and WHtR (β = 0.005, *p* < 0.01), while higher Daytime Sleepiness scores were negatively associated with both BMI (β = −0.056, *p* < 0.01) and WHtR (β = −0.001, *p* < 0.05). Higher Sleep Duration scores were negatively associated with BMI (β = −0.067, *p* < 0.05). In logistic regression models, Sleep Disordered Breathing was significantly associated with higher odds of both overweight/obesity (OR = 1.39, 95% CI: 1.13–1.70), and central obesity (OR = 1.36, 95% CI: 1.11–1.68). These associations were more evident among boys.

**Conclusion:**

Different dimensions of sleep problems showed varying associations with overweight/obesity and central obesity in children, with stronger associations observed among boys.

## 1 Introduction

Childhood and adolescent obesity has become a global public health concern. Recent estimates indicate that 14.8% of children and adolescents worldwide are overweight, and 8.5% have obesity. Notably, the prevalence of obesity is higher among preschool-aged (8.46%) and school-aged children (9.36%) compared to adolescents (6.92%) (1). In China, the prevalence of overweight and obesity among students has increased over the past decades, varying by age, sex, and region, with the highest rates observed in primary school students (2). A recent study reported that 15.8% of Chinese children and adolescents aged 3–18 are overweight, and 8.9% are living with obesity (3).

Sleep health encompasses multiple dimensions of sleep–wake patterns. Sleep is a fundamental psychophysiological process, and sufficient, high-quality sleep is essential for the healthy development of children and adolescents (4). The American Academy of Sleep Medicine recommends 9–12 h of sleep per day for children aged 6–12, and 8–10 h for adolescents aged 13–18 (5). In China, primary school students are advised to sleep at least 10 h per day, while middle and high school students should obtain at least 9 and 8 h, respectively (6). However, it is noteworthy that insufficient sleep is common among children and adolescents. A systematic review of data from 20 countries found a consistent decline in sleep duration among children and adolescents aged 5–18 from 1905 to 2008 (7). A survey conducted in China reported that 74.0% of children aged 6–11 and 71.2% of those aged 12–14 experience inadequate sleep (8).

Importantly, poor sleep quality has been associated with key indicators of physical health (9). Body mass index (BMI), waist circumference, and body fat percentage are key indicators of physical health in children and adolescents and are widely used in clinical assessments (10). Meta-analyses have shown that longer sleep duration, better sleep quality, and fewer insomnia symptoms are associated with lower BMI and body fat percentage (9). Sex-specific differences have also been observed in the association between sleep duration and BMI (11). The waist-to-height ratio (WHtR) is another useful index that reflects visceral fat accumulation. A school-based cross-sectional study in 2016 among Chinese adolescents found that insufficient sleep in those aged 12–13 was significantly associated with higher BMI, hip circumference, waist circumference, and WHtR (12). Similar to BMI, the association between sleep duration and WHtR may also differ by sex (13).

Although numerous studies have examined the relationship between sleep duration and obesity or anthropometric indicators in children and adolescents, relatively few have focused on sleep problems. By investigating third-grade primary school students in Shanghai, this study provides new insights into the associations between sleep problems and BMI, WHtR, overweight/obesity, and central obesity. Furthermore, it addresses the limited evidence on sex-specific associations with body composition, as measured by these anthropometric indicators.

## 2 Methods

### 2.1 Study design and subjects

The participants in this study were third-grade primary school students enrolled in the Shanghai Municipal Dynamic Cohort of Student Common Diseases (SMDCSCD) (14, 15). Since 2021, a multi-stage stratified cluster sampling method has been used to recruit a representative sample from schools across all 16 districts of Shanghai, with each district contributing one primary school and one junior high school. Participants are followed up annually, and the data for this study were derived from the 2023 follow-up of third-grade primary school students. The research objectives were communicated to school administrators and teachers, and after obtaining official approval, anthropometric measurements were conducted for eligible students. Meanwhile, their parents completed an electronic questionnaire explaining the study purpose. In total, 3,640 students aged 8–10 years were surveyed. Of these, 16 students were excluded due to missing data in the Children’s Sleep Habits Questionnaire (CSHQ). An additional 509 students were excluded due to missing or abnormal values in key covariates, including sex, parental educational level, waist circumference, parental BMI, daily screen time, and daily outdoor activity time. Ultimately, 3,115 students with complete data were included in the final analysis. Electronic informed consent was obtained from the parents or legal guardians of all participants. The study protocol was approved by the Ethics Review Committee of the Shanghai Municipal Center for Disease Control and Prevention.

### 2.2 Anthropometric measurements

Anthropometric measurements were conducted at school by trained research personnel using standardized procedures and calibrated instruments. Height and weight were measured following standard protocols, with participants wearing light clothing, standing upright, and barefoot. Height was recorded to the nearest 0.1 cm, and weight to the nearest 0.1 kg. Waist circumference was measured to the nearest 0.1 cm at the midpoint between the lower margin of the last rib and the iliac crest at the end of a normal exhalation. BMI was calculated as weight in kilograms divided by the square of height in meters (kg/m^2^). WHtR was computed by dividing waist circumference (cm) by height (cm).

### 2.3 Socio-demographic questionnaire

Socio-demographic data were collected using a structured questionnaire completed by the students’ parents. The questionnaire covered information on district, school, grade, class, sex, parental education level, parental height and weight, physical activity, dietary habits, sleep patterns, and other relevant characteristics. Completed questionnaires were reviewed by trained personnel and entered into a database.

The CSHQ is a validated instrument originally developed by Owens et al. (16) to screen for behaviorally and medically based sleep problems in school-aged children. Since its development, the CSHQ has been translated into multiple languages and is widely applied in diverse populations across countries. In this study, we used the Chinese version of the CSHQ, which was developed through a standardized process of translation and back-translation, and has demonstrated high sensitivity and reliability in Chinese populations (17–19).

The CSHQ consists of two main components: sleep duration and sleep-related problems. Parents reported their child’s typical bedtime and wake time on both weekdays and weekends over the past week. Average sleep duration was calculated as a weighted average: [(weekday sleep duration × 5) + (weekend sleep duration × 2)] / 7. Sleep problems were assessed based on 32 items rated on a three-point Likert scale: “frequently” (5–7 times per week), “occasionally” (2–4 times per week), and “rarely” (0–1 times per week). These items cover eight specific domains: Bedtime Resistance, Sleep Anxiety, Sleep Duration, Sleep Disordered Breathing, Parasomnias, Daytime Sleepiness, Night Wakings, and Sleep Onset Delay (17, 18). Subscale and total scores were computed according to the standard scoring method, with higher scores indicating more severe sleep problems. In the original study, a cutoff score of >41 was identified as the most sensitive threshold for detecting sleep problems (16, 19), and this value has been widely adopted in Chinese studies as well.

### 2.4 Statistical analysis

Descriptive statistics were reported as means and standard deviations (SD) for continuous variables, and as percentages for categorical variables. Group differences were assessed using independent-sample *t*-tests for continuous variables and χ^2^ tests for categorical variables, as appropriate.

Multiple linear regression models were used to examine the associations between anthropometric indicators (BMI and WHtR) and sleep variables, including average sleep duration, weekday and weekend sleep duration, total CSHQ score, and the eight subscale scores. Models were adjusted for relevant covariates. Students were classified as having overweight or obesity, or neither, based on BMI, according to the national standard *Screening for Overweight and Obesity among School-age Children and Adolescents* (WS/T 586-2018) issued by the National Health Commission of China (20, 21). For convenience, the term “overweight/obesity” is used throughout this manuscript to refer to this combined category. Similarly, central obesity was defined using a WHtR cutoff of ≥0.46, which has shown good performance in identifying excess abdominal fat and associated cardiometabolic risks in children (22, 23). Based on this threshold, students were categorized as having or not having central obesity.

Logistic regression models were applied to estimate the associations of insufficient sleep and sleep problems with overweight/obesity and central obesity. In these models, a value of “1” indicated overweight/obesity or central obesity, and “0” indicated non-overweight/obesity or non-central obesity, respectively. In line with Chinese guidelines recommending at least 10 h of sleep per day for primary school students, insufficient sleep was defined as an average sleep duration of less than 10 h. All sleep variables were first evaluated in univariate models, followed by multivariate models adjusting for sociodemographic and lifestyle covariates. Covariates included sex (boys/girls), parental weight status (overweight/obesity vs. non- overweight/obesity), parental educational level (below college/university vs. college/university and above), daily screen time (≤1 h, 1–2 h, >2 h), daily outdoor activity time (≤1 h, 1–2 h, >2 h), and weekly fried food intake frequency (0 times, 1–2 times, ≥3 times).

All statistical analyses were conducted in R (version 4.4.2; R Foundation for Statistical Computing, Vienna, Austria). A two-tailed *p* value < 0.05 was considered statistically significant.

## 3 Results

### 3.1 Descriptive analysis

Table 1 presents the sociodemographic and anthropometric characteristics of the participants. The mean age of the students was 8.6 ± 0.3 years. The average height, weight, waist circumference, BMI, and WHtR were 133.8 ± 6.0 cm, 30.8 ± 7.0 kg, 58.5 ± 7.9 cm, 17.1 ± 3.0 kg/m^2^, and 0.44 ± 0.05, respectively. The prevalence of overweight/obesity was 31.7%, with a significantly higher rate among boys (39.4%) than girls (23.9%). The prevalence of central obesity was 27.8%, with a significantly higher rate among boys (36.5%) than girls (19.0%).

**Table 1:** Sociodemographic and anthropometric characteristics of the sample (*n* = 3,115).

Variables	Boys (*n* = 1,574)	Girls (*n* = 1,541)	*P* value
Age (years, mean ± SD)	8.62 ± 0.30	8.61 ± 0.30	0.188
Height (cm, mean ± SD)	134.43 ± 6.01	133.10 ± 6.00	<0.001
Weight (kg, mean ± SD)	32.25 ± 7.39	29.26 ± 6.31	<0.001
Waist circumference (cm, mean ± SD)	60.59 ± 8.59	56.45 ± 6.49	<0.001
BMI (kg/m^2^, mean ± SD)	17.71 ± 3.15	16.42 ± 2.78	<0.001
WHtR (mean ± SD)	0.45 ± 0.06	0.42 ± 0.04	<0.001
BMI classification, *n* (%)			<0.001
Non-overweight/obesity	954 (60.6)	1,173 (76.1)	
Overweight/obesity	620 (39.4)	368 (23.9)	
WHtR classification, *n* (%)			<0.001
Non-central obesity	1,000 (63.5)	1,248 (81.0)	
Central obesity	574 (36.5)	293 (19.0)	
Father’s education level, *n* (%)			0.453
Below college/university	229 (14.5)	240 (15.6)	
College/university and above	1,345 (85.5)	1,301 (84.4)	
Mother’s education level, *n* (%)			0.391
Below college/university	244 (15.5)	221 (14.3)	
College/university and above	1,330 (84.5)	1,320 (85.7)	
Father’s weight status, *n* (%)			0.635
Non-overweight/obesity	719 (45.7)	718 (46.6)	
Overweight/obesity	855 (54.3)	823 (53.4)	
Mother’s weight status, *n* (%)			0.634
Non-overweight/obesity	1,289 (81.9)	1,273 (82.6)	
Overweight/obesity	285 (18.1)	268 (17.4)	
Daily screen time, *n* (%)			0.114
≤1 h	1,122 (71.3)	1,147 (74.4)	
1–2 h	366 (23.3)	326 (21.2)	
>2 h	86 (5.5)	68 (4.4)	
Daily outdoor activity time, *n* (%)			0.227
≤1 h	767 (48.7)	797 (51.7)	
1–2 h	656 (41.7)	611 (39.6)	
>2 h	151 (9.6)	133 (8.6)	
Fried food intake/week, *n* (%)			0.095
0 times	737 (46.8)	757 (49.1)	
1–2 times	739 (47.0)	713 (46.3)	
≥3 times	98 (6.2)	71 (4.6)	

BMI, body mass index; SD, standard deviation; WHtR, waist-to-height ratio; h, hour; cm, centimeter; kg, kilogram.

*p* values were calculated using independent-samples *t* test for continuous variables and chi-squared test for categorical variables.

Table 2 summarizes the sleep characteristics of the participants. The average sleep duration was 9.2 ± 0.6 h, with 9.0 ± 0.6 h on weekdays and 9.7 ± 0.8 h on weekends. Girls had significantly longer average and weekend sleep durations compared to boys (*p* < 0.05). The mean total score of CSHQ was 47.8 ± 7.5. Using a cutoff of 41, 79.7% of students were identified as having sleep problems, with a significantly higher proportion in girls than in boys (*p* < 0.05). The proportions of students with potential problems in the eight CSHQ subscales were as follows: Bedtime Resistance (97.0%), Sleep Anxiety (62.0%), Sleep Duration (94.9%), Sleep Disordered Breathing (16.9%), Parasomnias (41.0%), Daytime Sleepiness (67.8%), Night Wakings (15.8%), and Sleep Onset Delay (95.0%). Among these, the prevalence of Sleep Disordered Breathing was significantly higher in boys than in girls (*p* < 0.01). It should be noted that being classified as “abnormal” does not imply a clinical diagnosis, but rather a higher likelihood of difficulties in the respective sleep domain.

**Table 2:** Sleep characteristics of the sample (*n* = 3,115).

Variables	Boys (*n* = 1,574)	Girls (*n* = 1,541)	*P* value
Weekday sleep duration (h, mean ± SD)	9.01 ± 0.65	9.02 ± 0.64	0.643
Weekend sleep duration (h, mean ± SD)	9.62 ± 0.77	9.76 ± 0.77	<0.001
Average sleep duration (h, mean ± SD)	9.19 ± 0.60	9.23 ± 0.59	<0.05
Sleep sufficiency, *n* (%)			
Sufficient (≥10 h)	106 (6.7)	128 (8.3)	0.110
Insufficient (<10 h)	1,468 (93.3)	1,413 (91.7)	
CSHQ total score (mean ± SD)	47.53 ± 7.47	48.01 ± 7.45	0.070
CSHQ-defined sleep problems, *n* (%)			<0.05
No (CSHQ score ≤ 41)	346 (22.0)	286 (18.6)	
Yes (CSHQ score > 41)	1,228 (78.0)	1,255 (81.4)	
Bedtime resistance, *n* (%)			0.918
Normal	49 (3.1)	46 (3.0)	
Abnormal	1,525 (96.9)	1,495 (97.0)	
Sleep anxiety, *n* (%)			0.057
Normal	624 (39.6)	559 (36.3)	
Abnormal	950 (60.4)	982 (63.7)	
Sleep duration (CSHQ subscale), *n* (%)			1.000
Normal	80 (5.1)	78 (5.1)	
Abnormal	1,494 (94.9)	1,463 (94.9)	
Sleep-disordered breathing, *n* (%)			<0.01
Normal	1,276 (81.1)	1,313 (85.2)	
Abnormal	298 (18.9)	228 (14.8)	
Parasomnias, *n* (%)			0.812
Normal	932 (59.2)	905 (58.7)	
Abnormal	642 (40.8)	636 (41.3)	
Daytime sleepiness, *n* (%)			0.524
Normal	498 (31.6)	505 (32.8)	
Abnormal	1,076 (68.4)	1,036 (67.2)	
Night wakings, *n* (%)			0.875
Normal	1,328 (84.4)	1,296 (84.1)	
Abnormal	246 (15.6)	245 (15.9)	
Sleep onset delay, *n* (%)			0.148
Normal	70 (4.4)	87 (5.6)	
Abnormal	1,504 (95.6)	1,454 (94.4)	

CSHQ, Children’s Sleep Habits Questionnaire; SD, standard deviation; h, hour.

*p* values were calculated by chi-squared tests for categorical variables and independent-samples *t* test for continuous variables.

### 3.2 Associations between sleep problems and BMI

Multiple linear regression analysis was performed to assess the association between sleep variables and BMI (Table 3). After adjusting for covariates, no statistically significant association was observed between BMI and average sleep duration (*p* = 0.097). However, each additional hour of weekend sleep was associated with a 0.188-unit decrease in BMI (*p* < 0.01). Higher scores on specific CSHQ subscales were also significantly associated with BMI: each one-point increase in the Sleep Disordered Breathing score was associated with a 0.246-unit increase in BMI (*p* < 0.01); each point increase in the Sleep Duration score was associated with a 0.067-unit decrease in BMI (*p* < 0.05); and each point increase in the Daytime Sleepiness score was associated with a 0.056-unit decrease in BMI (*p* < 0.01).

**Table 3:** Association between sleep problems indicators and BMI by multiple linear regression (*n* = 3,115).

Sleep variables	Unadjusted model		Adjusted model	
	β (95% CI)	*P* value	β (95% CI)	*P* value
Average sleep duration (h)	−0.222 (−0.400, −0.043)	<0.05	−0.143 (−0.312, 0.026)	0.097
Weekday sleep duration (h)	−0.106 (−0.272, 0.059)	0.209	−0.064 (−0.220, 0.092)	0.421
Weekend sleep duration (h)	−0.279 (−0.417, −0.140)	<0.01	−0.188 (−0.319, −0.057)	<0.01
CSHQ total score	−0.014 (−0.028, 0.001)	0.064	−0.013 (−0.026, 0.001)	0.069
Bedtime resistance (score)	−0.029 (−0.070, 0.011)	0.152	−0.020 (−0.058, 0.018)	0.293
Sleep anxiety (score)	−0.010 (−0.062, 0.041)	0.700	−0.004 (−0.052, 0.045)	0.883
Sleep duration (score, CSHQ subscale)	−0.053 (−0.121, 0.015)	0.128	−0.067 (−0.131, −0.003)	<0.05
Sleep disordered breathing (score)	0.303 (0.137, 0.469)	<0.01	0.246 (0.090, 0.402)	<0.01
Parasomnias (score)	−0.052 (−0.120, 0.017)	0.142	−0.043 (−0.107, 0.022)	0.197
Daytime sleepiness (score)	−0.055 (−0.098, −0.013)	<0.05	−0.056 (−0.096, −0.015)	<0.01
Night wakings (score)	−0.004 (−0.147, 0.139)	0.954	0.012 (−0.123, 0.146)	0.864
Sleep onset delay (score)	−0.084 (−0.277, 0.110)	0.396	−0.096 (−0.278, 0.086)	0.301

CSHQ, Children’s Sleep Habits Questionnaire; BMI, body mass index; CI, confidence interval; h, hour.

The adjusted model was controlled for sex (boys, girls), parental BMI, parental education level (below college/university, college/university and above), daily screen time (h), daily outdoor activity time (h), and weekly fried food intake frequency (0 times, 1–2 times, ≥3 times).

Sex-stratified models indicated that, among boys, BMI was negatively associated with weekend sleep duration (β = −0.273, *p* < 0.01), CSHQ total score (β = −0.027, *p* < 0.01), Sleep Duration (β = −0.190, *p* < 0.01), and Daytime Sleepiness (β = −0.111, *p* < 0.01). Sleep Disordered Breathing was positively correlated with BMI in boys (β = 0.343, *p* < 0.01). No significant associations were found between sleep indicators and BMI among girls (Supplementary Table S1).

### 3.3 Associations between sleep problems and WHtR

The same analytical approach was applied to assess the associations between sleep variables and WHtR (Table 4). After adjusting for covariates, higher scores on specific subscales were significantly associated with WHtR: a one-point increase in the Sleep Disordered Breathing score was associated with a 0.005-unit increase in WHtR (*p* < 0.01), while each point increase in the Daytime Sleepiness score was associated with a 0.001-unit decrease (*p* < 0.05). No significant associations were observed between WHtR and average sleep duration (*p* = 0.062), weekday sleep duration (*p* = 0.156), or weekend sleep duration (*p* = 0.097).

**Table 4:** Association between sleep problems and WHtR by multiple linear regression (*n* = 3,115).

Sleep variables	Unadjusted model		Adjusted model	
	β (95% CI)	*P* value	β (95% CI)	*P* value
Average sleep duration (h)	−0.004 (−0.008, −0.001)	<0.01	−0.003 (−0.006, 0.000)	0.062
Weekday sleep duration (h)	−0.003 (−0.006, −0.000)	<0.05	−0.002 (−0.005, 0.000)	0.156
Weekend sleep duration (h)	−0.004 (−0.006, −0.001)	<0.01	−0.002 (−0.004, 0.000)	0.097
CSHQ total score	−0.000 (−0.000, 0.000)	0.411	−0.000 (−0.000, 0.000)	0.468
Bedtime resistance (score)	−0.000 (−0.001, 0.001)	0.711	0.000 (−0.001, 0.001)	0.853
Sleep anxiety (score)	−0.000 (−0.001, 0.001)	0.886	0.000 (−0.001, 0.001)	0.778
Sleep duration (score, CSHQ subscale)	−0.000 (−0.002, 0.001)	0.483	−0.001 (−0.002, 0.000)	0.194
Sleep disordered breathing (score)	0.006 (0.003, 0.009)	<0.01	0.005 (0.002, 0.008)	<0.01
Parasomnias (score)	−0.001 (−0.002, 0.001)	0.388	−0.000 (−0.002, 0.001)	0.472
Daytime sleepiness (score)	−0.001 (−0.002, 0.000)	<0.05	−0.001 (−0.002, 0.000)	<0.05
Night wakings (score)	0.001 (0.000, 0.004)	0.352	0.001 (−0.001, 0.004)	0.264
Sleep onset delay (score)	−0.000 (−0.004, 0.003)	0.809	−0.001 (−0.004, 0.002)	0.568

CSHQ, Children’s Sleep Habits Questionnaire; WHtR, waist-to-height ratio; CI, confidence interval; h, hour.

The adjusted model was controlled for sex (boys, girls), parental BMI, parental education level (below college/university, college/university and above), daily screen time (h), daily outdoor activity time (h), and weekly fried food intake frequency (0 times, 1–2 times, ≥3 times).

In sex-stratified models, negative associations with WHtR were observed in boys for weekend sleep duration (β = −0.005, *p* < 0.01) and Daytime Sleepiness (β = −0.002, *p* < 0.01). Sleep Disordered Breathing was positively associated with WHtR in boys (β = 0.007, *p* < 0.01). No significant associations were found between sleep indicators and WHtR among girls (Supplementary Table S2).

### 3.4 Associations between sleep problems and overweight/obesity in students

The results of the logistic regression analysis examining the association between sleep problems and overweight/obesity are presented in Table 5. In the adjusted model, students with probable Sleep Disordered Breathing had 1.39 times higher odds of being overweight or obesity compared to those without (95% CI: 1.13–1.70; *p* < 0.01).

**Table 5:** Association between sleep problems and overweight/obesity by logistic regression (*n* = 3,115).

Sleep variables	Unadjusted model		Adjusted model	
	OR (95% CI)	*P* value	OR (95% CI)	*P* value
Average sleep duration (insufficient)	0.94 (0.71, 1.25)	0.685	0.91 (0.67, 1.22)	0.527
Sleep problems (yes)	0.93 (0.77, 1.11)	0.413	0.94 (0.78, 1.15)	0.563
Bedtime resistance (abnormal)	0.79 (0.52, 1.21)	0.277	0.80 (0.51, 1.26)	0.336
Sleep anxiety (abnormal)	0.93 (0.80, 1.08)	0.350	0.96 (0.81, 1.12)	0.582
Sleep duration (abnormal)	0.86 (0.62, 1.21)	0.391	0.91 (0.64, 1.30)	0.612
Sleep disordered breathing (abnormal)	1.47 (1.21, 1.79)	<0.01	1.39 (1.13, 1.70)	<0.01
Parasomnias (abnormal)	0.87 (0.74, 1.01)	0.068	0.86 (0.73, 1.01)	0.061
Daytime sleepiness (abnormal)	0.88 (0.75, 1.03)	0.120	0.86 (0.73, 1.02)	0.081
Night wakings (abnormal)	1.01 (0.82, 1.25)	0.893	1.01 (0.81, 1.25)	0.961
Sleep onset delay (abnormal)	1.33 (0.93, 1.92)	0.123	1.34 (0.92, 1.97)	0.131

OR, odds ratio; CI, confidence interval.

Values in parentheses next to sleep variables indicate the exposure categories. The adjusted model was controlled for sex (boys, girls), parental weight status (overweight/obesity, non-overweight/obesity), parental education level (below college/university, college/university and above), daily screen time (≤1 h, 1–2 h, >2 h), daily outdoor activity time (≤1 h, 1–2 h, >2 h), and weekly fried food intake frequency (0 times, 1–2 times, ≥3 times).

In sex-stratified models, boys with probable Sleep Disordered Breathing had increased odds of overweight/obesity (OR = 1.48, 95% CI: 1.14–1.93; *p* < 0.01). Additionally, boys with probable Daytime Sleepiness had lower odds of overweight/obesity (OR = 0.74; 95% CI: 0.59–0.92; *p* < 0.01). No significant associations were found between sleep indicators and overweight/obesity among girls (Supplementary Table S3).

### 3.5 Associations between sleep problems and central obesity in students

The results of the logistic regression analysis examining the association between sleep problems and central obesity are presented in Table 6. In the adjusted model, students with probable Sleep Disordered Breathing had 1.36 times higher odds of central obesity compared to those without (95% CI: 1.11–1.68; *p* < 0.01). Students with probable Daytime Sleepiness had 0.83 times lower odds of central obesity compared to those without (95% CI: 0.70–0.99; *p* < 0.05).

**Table 6:** Association between sleep problems and central obesity by logistic regression (*n* = 3,115).

Sleep variables	Unadjusted model		Adjusted model	
	OR (95% CI)	*P* value	OR (95% CI)	*P* value
Average sleep duration (insufficient)	1.00 (0.74, 1.35)	0.984	0.99 (0.72, 1.35)	0.934
Sleep problems (yes)	0.91 (0.75, 1.11)	0.366	0.94 (0.77, 1.15)	0.542
Bedtime resistance (abnormal)	0.68 (0.45, 1.05)	0.081	0.74 (0.47, 1.16)	0.185
Sleep anxiety (abnormal)	0.96 (0.81, 1.12)	0.579	1.00 (0.85, 1.19)	0.982
Sleep duration (abnormal)	0.67 (0.48, 0.93)	<0.05	0.72 (0.51, 1.03)	0.073
Sleep disordered breathing (abnormal)	1.47 (1.20, 1.80)	<0.01	1.36 (1.11, 1.68)	<0.01
Parasomnias (abnormal)	0.87 (0.74, 1.02)	0.093	0.86 (0.73, 1.02)	0.079
Daytime sleepiness (abnormal)	0.84 (0.71, 0.99)	<0.05	0.83 (0.70, 0.99)	<0.05
Night wakings (abnormal)	1.08 (0.87, 1.33)	0.487	1.06 (0.85, 1.33)	0.584
Sleep onset delay (abnormal)	1.26 (0.87, 1.84)	0.222	1.30 (0.87, 1.93)	0.202

OR, odds ratio; CI, confidence interval.

Values in parentheses next to sleep variables indicate the exposure categories. Central obesity was defined as a waist-to-height ratio (WHtR) ≥ 0.46. The adjusted model was controlled for sex (boys, girls), parental weight status (overweight/obesity, non-overweight/obesity), parental education level (below college/university, college/university and above), daily screen time (≤1 h, 1–2 h, >2 h), daily outdoor activity time (≤1 h, 1–2 h, >2 h), and weekly fried food intake frequency (0 times, 1–2 times, ≥3 times).

In sex-stratified models, boys with probable Sleep Disordered Breathing had increased odds of central obesity (OR = 1.43, 95% CI: 1.10–1.86; *p* < 0.01). Additionally, boys with probable Daytime Sleepiness had lower odds of central obesity (OR = 0.79; 95% CI: 0.63–0.99; *p* < 0.05). No significant associations were found between sleep indicators and central obesity among girls (Supplementary Table S4).

## 4 Discussion

This study recruited a representative sample of third-grade students (mean age 8.6 ± 0.3 years) from primary schools in Shanghai, and evaluated their sleep problems using the CSHQ. The findings revealed that longer weekend sleep duration was significantly associated with lower BMI. Higher scores in the Sleep Disordered Breathing subscale were positively associated with both BMI and WHtR, while higher Daytime Sleepiness scores were negatively correlated with these indicators. Additionally, elevated Sleep Duration subscale scores were also inversely associated with BMI. Children with Sleep Disordered Breathing also had significantly increased odds of being overweight/obesity, as well as of having central obesity. Notably, these associations appeared stronger in boys.

The mean sleep duration observed in this study was 9.2 h, which falls below the recommended 10 h for primary school children in China. This finding aligns with a large-scale national study reporting an average sleep duration of 9.31 h for Chinese children aged 9.35 years (24). However, children in our sample slept notably less than their Western peers. For example, Mexican-American children aged 8.8 years reportedly averaged 9.6 h of sleep (25). Cross-national studies indicate that Chinese children tend to go to bed later, wake up earlier, and sleep approximately 1 h less than children in the United States (26). National surveys have shown that 13.3% of Chinese children aged 6–12 years do not get enough sleep on school days, and 4.6% experience insufficient sleep even on weekends (27). Our data also demonstrate that children extend their sleep duration on weekends, reflecting a disrupted and irregular sleep pattern. Prior research suggests that irregular weekly sleep schedules may lead to persistent fatigue, cognitive impairments, and behavioral dysfunction resembling jet lag (28). Moreover, weekend catch-up sleep fails to fully reverse the adverse effects of insufficient weekday sleep, including impairments in executive function (29). These irregular patterns may interfere with circadian-regulated physiological processes, such as the secretion of melatonin, cortisol, and growth hormone, as well as immune function via cytokine rhythms (30, 31).

Previous studies have reported that longer sleep duration is inversely associated with BMI and other adiposity indicators (24, 32). For instance, a cross-sectional study in China found that short sleep was associated with higher BMI, greater waist circumference, and elevated WHtR among 9–12-year-olds (33). Experimental research has shown that sleep restriction can increase responsiveness of reward pathways to food cues and promote visceral fat accumulation (34, 35). However, in our study, average sleep duration was not significantly associated with BMI, WHtR, or the odds of overweight/obesity after adjusting for potential confounders. This finding is consistent with findings from Canadian, Australian, as well as a Peruvian study, all of which failed to detect a robust relationship between sleep duration and obesity after multivariable adjustment (36–38). Differences in study design, population characteristics, age range, and adjustment strategies may account for these inconsistent findings.

Interestingly, our study found a significant inverse association between weekend sleep duration and BMI, while no such association was observed during weekdays. Similar results have been reported among children aged 7–14 years, where weekday sleep duration was not linked to obesity risk (39). This may be explained by the uniformly restricted and structured sleep schedules during weekdays due to academic demands and parental regulation, whereas longer sleep on weekends may act as a compensatory mechanism. Indeed, children who failed to catch up on sleep during weekends were more likely to be overweight or obese than those who did (40, 41). The metabolic consequences of insufficient and irregular sleep—including insulin resistance, inflammation, and disrupted energy balance—could underlie these associations (42). Nonetheless, the benefits of weekend compensation may be limited, as irregular sleep schedules can impair overall sleep quality and daytime functioning (43). Notably, sex differences in sleep-obesity associations have also been observed. Our findings align with those of a French study showing that girls sleep longer than boys on non-school days, with no differences on weekdays (44). Moreover, studies from Korea indicate that reduced sleep duration is significantly associated with obesity indices in boys, but not in girls (13). These disparities may reflect underlying differences in lifestyle, hormonal profiles, and social influences between sexes.

The proportion of children classified as having sleep problems in our study was 79.7%, which is comparable to previous findings. For example, a U. S. study reported that 94% of minority children aged 5–6 years exceeded the CSHQ cut-off score of 41 (45), while approximately 80% of Japanese preschoolers and 62.4% of school-aged children in a Japanese community sample also surpassed this threshold (46, 47). Similarly, a nationally representative study in China found that 76.78% of preschool-aged children exhibited sleep problems based on the CSHQ (48, 49). It is important to note that the CSHQ is a screening tool rather than a diagnostic instrument, and the commonly used threshold of >41 is intended to flag potential sleep disturbances rather than indicate clinical disorders. Children’s sleep behaviors are influenced by a combination of biological, psychological, cultural, and societal factors. The >41 cut-off was originally validated in Western populations, and its cross-cultural applicability has been questioned. Differences in parenting practices, sleep expectations, and symptom perception may influence how parents rate items. A Chinese study exploring the link between sleep and behavioral problems highlighted concerns about the >41 cutoff and conducted a sensitivity analysis using a higher threshold of 48, which yielded comparable results (48). Some researchers have suggested that future studies adopt culturally sensitive and comparable approaches to explore the reasons behind cultural differences in children’s sleep. They also recommended evaluating the need to develop age- and sex-specific norms for the CSHQ (49). Therefore, the generalizability of this 41-threshold across cultures should be interpreted with caution. Further studies are needed to establish culturally adapted cutoff values in Chinese children.

Sleep quality plays a crucial role in metabolic regulation. Poor sleep quality has been linked to increased BMI and adiposity, independent of sleep duration (9). Mechanistically, disrupted sleep quality alters substrate oxidation, leptin and ghrelin levels, resting metabolic rate, appetite regulation, reward-related food processing, hypothalamic–pituitary–adrenal axis activity, and gut-peptide secretion, ultimately promoting positive energy balance and weight gain (50). Conversely, obesity itself can worsen sleep quality, establishing a bidirectional and self-reinforcing relationship between poor sleep and weight gain (51).

In our study, Sleep Disordered Breathing (SDB) score was positively associated with both BMI and WHtR, consistent with previous findings (52). Epidemiological studies have shown that the prevalence of SDB is significantly higher among children with obesity (20.8%) than their normal-weight peers (6.3%) (53). This association is partly explained by obesity-induced changes in upper airway anatomy and pulmonary function (54). One type of SDB, obstructive sleep apnea (OSA), has been shown to exacerbate weight gain through various mechanisms, may further contribute to weight gain through reduced energy expenditure, increased caloric intake, altered neurohormonal control of appetite, and sleep fragmentation (55). Interestingly, while the self-reported average sleep duration was not significantly associated with adiposity, the Sleep Duration subscale of the CSHQ was positively associated with BMI, underscoring the multidimensional nature of sleep assessment. Excessive daytime sleepiness (EDS) often arises as a consequence of sleep disorders (e.g., OSA, circadian misalignment), poor sleep hygiene, or comorbid conditions (56). Factors independently associated with EDS include waist circumference, anxiety/depression symptoms, sleep-onset difficulties, and asthma history (57). Among children with obesity, clinical studies have shown that SDB is associated with increased pro-inflammatory cytokines, elevated leptin levels, reduced adiponectin, and higher prevalence of EDS (58). However, in contrast to previous literature, our results showed a negative association between daytime sleepiness and both BMI and WHtR. Discrepancies may stem from population differences or assessment tools; we used the CSHQ subscale, while EDS is often measured using tools like the Epworth Sleepiness Scale (59). A comprehensive evaluation of sleepiness requires both subjective (e.g., sleep diaries and questionnaires) and objective (e.g., polysomnography and multiple sleep latency tests) assessments (56).

Sex-related neurophysiological differences may explain variations in sleep patterns and obesity risk. For example, boys exhibit more infralow brain activity during sleep, whereas girls show higher fast-frequency activity during wakefulness, reflecting earlier cortical maturation in females (60). In rodent models, estradiol promotes appropriate sleep–wake rhythms in females, with no comparable effect observed in males (61, 62). These sex differences are largely driven by estradiol’s effects on neural structures differentiated during fetal development (63). Population studies further support sex differences in sleep quality; for instance, Finnish girls exhibited shorter sleep latency, higher sleep efficiency, and less fragmentation than boys (64). These findings suggest that sex-specific recommendations for sleep duration and quality may be warranted (65).

Several limitations of this study should be noted. First, the cross-sectional design limits our ability to infer causal relationships between sleep characteristics and obesity indicators. Second, sleep-related data were based on parent-reported questionnaires and are therefore subject to recall bias. Objective measures such as actigraphy or polysomnography would provide more accurate assessments of sleep characteristics. Moreover, the CSHQ captures sleep behaviors over the previous week, which may not reflect habitual or long-term sleep patterns. Third, although the CSHQ is a widely used screening tool for pediatric sleep problems, it cannot substitute for clinical diagnoses based on standardized criteria, and the cultural validity of its cutoff values also warrants careful consideration. Lastly, due to the limitations of the questionnaire design, certain physiological covariates—such as pubertal status—were not collected, which limited our ability to adjust for their potential confounding effects.

## 5 Conclusion

In summary, among third-grade primary school students in Shanghai, both sleep duration and sleep problems—assessed via the CSHQ—were associated with BMI, WHtR, overweight/obesity, and central obesity, with notable sex-specific differences. These findings underscore the importance of considering multidimensional sleep characteristics and sex-based variations when addressing childhood obesity, and highlight the need for further research using objective sleep measures and longitudinal designs.

## Data Availability

The datasets presented in this article are not readily available because the data supporting this study are not publicly available due to ethical and privacy considerations, as they contain identifiable information about child participants. Data access may be granted upon reasonable request, subject to approval by the corresponding author and the institutional ethics committee. Requests to access the datasets should be directed to Lijing Sun, sunlijing@scdc.sh.cn.
