# Supplementary material for: Sleep problems and childhood adiposity: a cross-sectional study among third-grade students in Shanghai, China
Source: Front Public Health. 2025 Aug 14;13:1629048. doi: 10.3389/fpubh.2025.1629048 (PMC12390967; doi:10.3389/fpubh.2025.1629048)
Supplement: Supplementary file 1 [file Table_1.docx]

**Supplementary data**

**Table S1.** Association between sleep problems and BMI across sex categories by multiple linear regression

**Table S2.** Association between sleep problems and WHtR across sex categories by multiple linear regression

**Table S3.** Association between sleep problems and overweight/obesity across sex categories by logistic regression

**Table S4**. Association between sleep problems and central obesity across sex categories by logistic regression

**Table S1. Association between sleep problems and BMI across sex categories by multiple linear regression**

| Sleep variables | Boys (n =1,574) | | | | Girls (n = 1,541) | | | |
| --- | --- | --- | --- | --- | --- | --- | --- | --- |
|  | Unadjusted model | | Adjusted model | | Unadjusted model | | Adjusted model | |
|  | β (95% CI) | *P* value | β (95% CI) | *P* value | β (95% CI) | *P* value | β (95% CI) | *P* value |
| Average sleep duration (h) | -0.221(-0.479, 0.037) | 0.093 | -0.176(-0.426, 0.073) | 0.166 | -0.134(-0.369, 0.102) | 0.267 | -0.114(-0.343, 0.115) | 0.330 |
| Weekday sleep duration (h) | -0.105(-0.344, 0.134) | 0.388 | -0.060(-0.291, 0.172) | 0.614 | -0.090(-0.307, 0.127) | 0.418 | -0.069(-0.280, 0.141) | 0.519 |
| Weekend sleep duration (h) | -0.289(-0.491, -0.087) | <0.01 | -0.273(-0.468, -0.078) | <0.01 | -0.119(-0.300, 0.061) | 0.196 | -0.112(-0.287, 0.062) | 0.206 |
| CSHQ total score | -0.018(-0.039, 0.003) | 0.092 | -0.027(-0.047, -0.007) | <0.01 | -0.003(-0.022, 0.015) | 0.730 | 0.001(-0.017, 0.019) | 0.908 |
| Bedtime Resistance (score) | -0.031(-0.091, 0.029) | 0.304 | -0.048(-0.106, 0.010) | 0.106 | -0.005(-0.056, 0.047) | 0.863 | 0.003(-0.047, 0.052) | 0.914 |
| Sleep Anxiety (score) | 0.006(-0.070, 0.082) | 0.879 | -0.002(-0.075, 0.072) | 0.967 | -0.009(-0.075, 0.058) | 0.794 | -0.007(-0.071, 0.057) | 0.828 |
| Sleep Duration (score, CSHQ subscale) | -0.163(-0.262, -0.064) | <0.01 | -0.190(-0.287, -0.094) | <0.01 | 0.058(-0.029, 0.146) | 0.189 | 0.058(-0.026, 0.142) | 0.175 |
| Sleep Disordered Breathing (score) | 0.409(0.173, 0.644) | <0.01 | 0.343(0.116, 0.571) | <0.01 | 0.099(-0.123, 0.322) | 0.381 | 0.123(-0.090, 0.336) | 0.258 |
| Parasomnias (score) | -0.028(-0.128, 0.071) | 0.578 | -0.046(-0.142, 0.051) | 0.352 | -0.074(-0.164, 0.016) | 0.108 | -0.042(-0.129, 0.045) | 0.343 |
| Daytime Sleepiness (score) | -0.082(-0.144, -0.019) | <0.05 | -0.111(-0.172, -0.051) | <0.01 | -0.013(-0.069, 0.043) | 0.650 | -0.002(-0.056, 0.052) | 0.947 |
| Night Wakings (score) | 0.060(-0.144, 0.264) | 0.564 | 0.052(-0.145, 0.249) | 0.603 | -0.084(-0.274, 0.106) | 0.389 | -0.038(-0.220, 0.145) | 0.685 |
| Sleep Onset Delay (score) | -0.166(-0.454, 0.122) | 0.258 | -0.222(-0.501, 0.056) | 0.118 | 0.010(-0.235, 0.255) | 0.937 | 0.019(-0.217, 0.255) | 0.874 |

Abbreviations: CSHQ, Children’s Sleep Habits Questionnaire; BMI, body mass index; CI, confidence interval; h, hour.

Note: The adjusted model was controlled for parental BMI, parental education level (below college/university, college/university and above), daily screen time (h), daily outdoor activity time (h), and weekly fried food intake frequency (0 times, 1-2 times, ≥3 times).

**Table S2. Association between sleep problems and WHtR across sex categories by multiple linear regression**

| Sleep variables | Boys (n =1,574) | | | | Girls (n = 1,541) | | | |
| --- | --- | --- | --- | --- | --- | --- | --- | --- |
|  | Unadjusted model | | Adjusted model | | Unadjusted model | | Adjusted model | |
|  | β (95% CI) | *P* value | β (95% CI) | *P* value | β (95% CI) | *P* value | β (95% CI) | *P* value |
| Average sleep duration (h) | -0.005(-0.010, -0.001) | <0.05 | -0.004(-0.009, 0.000) | 0.066 | -0.002(-0.006, 0.002) | 0.330 | -0.001(-0.005, 0.003) | 0.542 |
| Weekday sleep duration (h) | -0.003(-0.008, 0.001) | 0.115 | -0.002(-0.007, 0.002) | 0.250 | -0.002(-0.006, 0.001) | 0.161 | -0.002(-0.005, 0.002) | 0.300 |
| Weekend sleep duration (h) | -0.005(-0.009, -0.001) | <0.01 | -0.005(-0.008, -0.001) | <0.01 | 0.000(-0.002, 0.003) | 0.763 | 0.001(-0.002, 0.004) | 0.603 |
| CSHQ total score | -0.000(-0.001, 0.000) | 0.315 | -0.000(-0.001, 0.000) | 0.080 | 0.000(-0.000, 0.000) | 0.499 | 0.000(-0.000, 0.000) | 0.298 |
| Bedtime Resistance (score) | -0.000(-0.001, 0.001) | 0.605 | -0.000(-0.002, 0.001) | 0.356 | 0.000(-0.000, 0.001) | 0.252 | 0.001(-0.000, 0.001) | 0.156 |
| Sleep Anxiety (score) | -0.000(-0.002, 0.001) | 0.562 | -0.000(-0.002, 0.001) | 0.583 | 0.001(-0.000, 0.002) | 0.253 | 0.001(-0.000, 0.002) | 0.231 |
| Sleep Duration (score, CSHQ subscale) | -0.001(-0.003, 0.001) | 0.239 | -0.002(-0.003, 0.000) | 0.064 | 0.000(-0.001, 0.002) | 0.703 | 0.000(-0.001, 0.002) | 0.771 |
| Sleep Disordered Breathing (score) | 0.008(0.004, 0.012) | <0.01 | 0.007(0.002, 0.011) | <0.01 | 0.003(-0.001, 0.006) | 0.136 | 0.003(-0.000, 0.007) | 0.075 |
| Parasomnias (score) | -0.000(-0.002, 0.001) | 0.738 | -0.001(-0.002, 0.001) | 0.470 | -0.001(-0.002, 0.001) | 0.338 | -0.000(-0.002, 0.001) | 0.753 |
| Daytime Sleepiness (score) | -0.001(-0.002, -0.000) | <0.05 | -0.002(-0.003, -0.001) | <0.01 | -0.000(-0.001, 0.001) | 0.915 | 0.000(-0.001, 0.001) | 0.931 |
| Night Wakings (score) | 0.002(-0.002, 0.006) | 0.314 | 0.002(-0.002, 0.005) | 0.365 | 0.000(-0.003, 0.003) | 0.881 | 0.001(-0.002, 0.004) | 0.519 |
| Sleep Onset Delay (score) | 0.001(-0.004, 0.006) | 0.769 | -0.001(-0.006, 0.004) | 0.811 | -0.001(-0.005, 0.003) | 0.552 | -0.001(-0.005, 0.003) | 0.509 |

Abbreviations: CSHQ, Children’s Sleep Habits Questionnaire; WHtR, waist-to-height ratio; CI, confidence interval; h, hour.

Note: The adjusted model was controlled for parental BMI, parental education level (below college/university, college/university and above), daily screen time (h), daily outdoor activity time (h), and weekly fried food intake frequency (0 times, 1-2 times, ≥3 times).

**Table S3. Association between sleep problems and overweight/obesity across sex categories by logistic regression**

| Sleep variables | Boys (n =1,574) | | | | Girls (n = 1,541) | | | |
| --- | --- | --- | --- | --- | --- | --- | --- | --- |
|  | Unadjusted model | | Adjusted model | | Unadjusted model | | Adjusted model | |
|  | OR (95% CI) | *P* value | OR (95% CI) | *P* value | OR (95% CI) | *P* value | OR (95% CI) | *P* value |
| Average sleep duration (insufficient) | 0.71 (0.48, 1.06) | 0.091 | 0.69 (0.45, 1.03) | 0.072 | 1.19 (0.76, 1.85) | 0.440 | 1.23 (0.77, 1.94) | 0.387 |
| Sleep quality (poor) | 0.96 (0.75, 1.22) | 0.736 | 0.94 (0.73, 1.21) | 0.634 | 0.96 (0.71, 1.30) | 0.794 | 0.95 (0.70, 1.29) | 0740 |
| Bedtime Resistance (abnormal) | 0.86 (0.49, 1.53) | 0.614 | 0.96 (0.53, 1.74) | 0.885 | 0.71 (0.37, 1.34) | 0.292 | 0.66 (0.34, 1.28) | 0.219 |
| Sleep Anxiety (abnormal) | 0.99 (0.81, 1.23) | 0.983 | 1.00 (0.81, 1.24) | 0.981 | 0.89 (0.70, 1.14) | 0.351 | 0.90 (0.70, 1.15) | 0.401 |
| Sleep Duration (abnormal) | 1.09 (0.68, 1.73) | 0.723 | 1.13 (0.70, 1.83) | 0.621 | 0.65 (0.40, 1.06) | 0.084 | 0.72 (0.43, 1.21) | 0.211 |
| Sleep Disordered Breathing (abnormal) | 1.55 (1.20, 1.99) | <0.01 | 1.48 (1.14, 1.93) | <0.01 | 1.23 (0.89, 1.69) | 0.204 | 1.26 (0.91, 1.75) | 0.171 |
| Parasomnias (abnormal) | 0.88 (0.71, 1.08) | 0.212 | 0.83 (0.67, 1.03) | 0.096 | 0.85 (0.67, 1.08) | 0.187 | 0.90 (0.70, 1.15) | 0.403 |
| Daytime Sleepiness (abnormal) | 0.77 (0.62, 0.95) | <0.05 | 0.74 (0.59, 0.92) | <0.01 | 1.03 (0.80, 1.32) | 0.839 | 1.05 (0.81, 1.36) | 0.730 |
| Night Wakings (abnormal) | 1.13 (0.86, 1.49) | 0.386 | 1.06 (0.80, 1.41) | 0.683 | 0.88 (0.64, 1.23) | 0.462 | 0.95 (0.68, 1.34) | 0.777 |
| Sleep Onset Delay (abnormal) | 1.35 (0.81, 2.24) | 0.254 | 1.46 (0.86, 2.47) | 0.159 | 1.22 (0.71, 2.07) | 0.473 | 1.17 (0.67, 2.04) | 0.586 |

Abbreviations: OR, odds ratio; CI, confidence interval.

Note: Values in parentheses next to sleep variables indicate the exposure categories. The adjusted model was controlled for parental weight status (overweight/obesity, non-overweight/obesity), parental education level (below college/university, college/university and above), daily screen time (≤1h, 1-2 h, >2 h), daily outdoor activity time (≤1 h, 1-2 h, >2 h), and weekly fried food intake frequency (0 times, 1-2 times, ≥3 times).

**Table S4. Association between sleep problems and central obesity across sex categories by logistic regression**

| Sleep variables | Boys (n =1,574) | | | | Girls (n = 1,541) | | | |
| --- | --- | --- | --- | --- | --- | --- | --- | --- |
|  | Unadjusted model | | Adjusted model | | Unadjusted model | | Adjusted model | |
|  | OR (95% CI) | *P* value | OR (95% CI) | *P* value | OR (95% CI) | *P* value | OR (95% CI) | *P* value |
| Average sleep duration (insufficient) | 0.80 (0.53, 1.19) | 0.265 | 0.80 (0.52, 1.21) | 0.280 | 1.21 (0.75, 1.98) | 0.433 | 1.27 (0.77, 2.11) | 0.347 |
| Sleep quality (poor) | 0.88 (0.69, 1.13) | 0.323 | 0.86 (0.67, 1.11) | 0.254 | 1.10 (0.79, 1.54) | 0.573 | 1.09 (0.77, 1.54) | 0.625 |
| Bedtime Resistance (abnormal) | 0.70 (0.39, 1.23) | 0.215 | 0.82 (0.46, 1.49) | 0.523 | 0.66 (0.34, 1.28) | 0.218 | 0.64 (0.32, 1.29) | 0.212 |
| Sleep Anxiety (abnormal) | 0.94 (0.76, 1.16) | 0.560 | 0.95 (0.77, 1.18) | 0.652 | 1.06 (0.81, 1.39) | 0.657 | **1.08 (0.82, 1.42)** | 0.572 |
| Sleep Duration (abnormal) | 0.73 (0.46, 1.14) | 0.166 | 0.78 (0.49, 1.25) | 0.311 | 0.58 (0.35, 0.96) | <0.05 | 0.67 (0.39, 1.13) | 0.134 |
| Sleep Disordered Breathing (abnormal) | 1.50 (1.16, 1.94) | <0.01 | 1.43 (1.10, 1.86) | <0.01 | 1.24 (0.88, 1.74) | 0.225 | 1.25 (0.88, 1.77) | 0.222 |
| Parasomnias (abnormal) | 0.84 (0.68, 1.04) | 0.107 | 0.79 (0.64, 0.99) | <0.05 | **0.92 (0.71, 1.19)** | 0.516 | 0.97 (0.74, 1.27) | 0.838 |
| Daytime Sleepiness (abnormal) | 0.78 (0.63, 0.98) | <0.05 | 0.79 (0.63, 0.99) | <0.05 | 0.88 (0.67, 1.15) | 0.334 | 0.89 (0.67, 1.18) | 0.421 |
| Night Wakings (abnormal) | 1.19 (0.90, 1.57) | 0.232 | 1.12 (0.84, 1.49) | 0.449 | 0.95 (0.67, 1.35) | 0.779 | 1.01 (0.70, 1.45) | 0.958 |
| Sleep Onset Delay (abnormal) | 1.10 (0.67, 1.83) | 0.698 | 1.23 (0.73, 2.07) | 0.432 | 1.36 (0.74, 2.48) | 0.321 | 1.33 (0.71, 2.48) | 0.369 |

Abbreviations: OR, odds ratio; CI, confidence interval.

Note: Values in parentheses next to sleep variables indicate the exposure categories. Central obesity was defined as a waist-to-height ratio (WHtR) ≥ 0.46. The adjusted model was controlled for parental weight status (overweight/obesity, non-overweight/obesity), parental education level (below college/university, college/university and above), daily screen time (≤1h, 1-2 h, >2 h), daily outdoor activity time (≤1 h, 1-2 h, >2 h), and weekly fried food intake frequency (0 times, 1-2 times, ≥3 times).
